# P35 ‘Antimicrobial Stewardship – Do It Right!’—a multidisciplinary clinical awareness-based quality improvement project

**DOI:** 10.1093/jacamr/dlac004.034

**Published:** 2022-02-16

**Authors:** Lee Xin, Duaa Ahmad

**Affiliations:** Barnsley Hospital NHS Foundation Trust, Barnsley, UK; Barnsley Hospital NHS Foundation Trust, Barnsley, UK

## Abstract

**Background:**

Based on a recent trust-wide antibiotic stewardship (AMS) audit, three specific AMS criteria were identified for improvement, namely appropriate sample collection before starting antibiotics, timely antibiotic reviews and documentation and microbiology discussions for restricted antibiotics.

**Objectives:**

To improve the performance of the aforementioned areas and assessing the efficacy of education materials and sessions in supporting AMS practice.

**Methods:**

Local and regional usage data, together with performance data from the audit were summarized into presentations, with teaching sessions organized for clinical staff over several months. Education materials via two different posters for prescribers, pharmacists, nurses and healthcare staff were distributed trust-wide in relevant clinical areas, together with on-site teaching and barrier analysis. Weekly run chart data of compliance to the three AMS criteria was collected from two inpatient wards (General Medicine and General Surgery) before and during the project to assess baseline compliance and measure project outcomes.

**Results:**

After 10 weeks, baseline AMS compliance achieved improvements of 5%–10% for appropriate sample collection (estimated baseline 60%), 20%–25% for timely antibiotic reviews and documentation (estimated baseline 65%) and 20%–25% for microbiology discussions of restricted antibiotics (estimated baseline 50%). Further data breakdown identified surgical specialities requiring additional AMS support, allowing for better allocation of microbiology resources, including a weekly ward round.

**Conclusions:**

Clinical awareness via a multidisciplinary approach remains key to improving AMS compliance, which can be improved and sustained effectively through regular staff education, either via teaching sessions or targeted education materials within relevant clinical areas.

